# Supplementary material for: A secreted effector with a dual role as a toxin and as a transcriptional factor
Source: Nat Commun. 2022 Dec 16;13:7779. doi: 10.1038/s41467-022-35522-9 (PMC9755527; doi:10.1038/s41467-022-35522-9)
Supplement: Supplementary file 1 — Supplementary Information [file 41467_2022_35522_MOESM1_ESM.pdf]

Supplementary Information

A secreted effector with a dual role as a toxin and as a transcriptional factor

This PDF file includes:

Supplementary Figures 1-13

Supplementary Tables 1

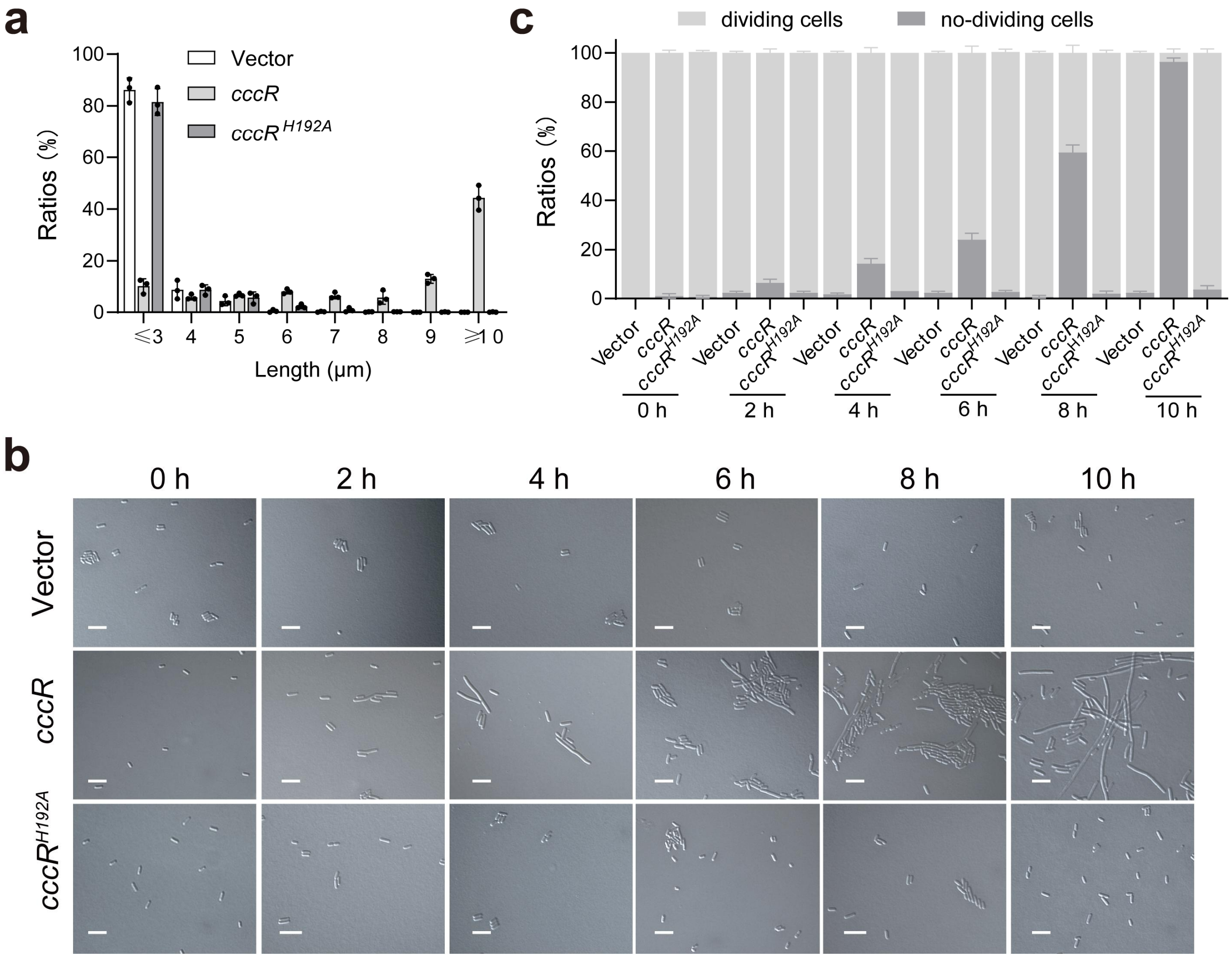

Supplementary Fig. 1 | CccR induces filamentation of *E. coli* cells.

a, Distribution of *cccR* expressing *E. coli* BL21(DE3) cells with different lengths. The length of 500 cells was measured from each of three samples, and their distribution was plotted. Data shown are representative from three independent experiments. **b-c**, CccR induces filamentation of *E. coli* cells. Representative micrographs of *E. coli* BL21(DE3) cells expressing CccR or CccR^{H192A} were acquired 0, 2, 4, 6, 8, and 10 h after induction of protein expression. Scale bar, 10 μm (**b**). Distribution of *cccR* expressing *E. coli* cells with different lengths at different time points (**c**). The length of 500 cells was measured from each of three samples, and their distribution was plotted. Data shown are representative from three independent experiments. Data in **a-c** are presented as the mean ± standard deviation (SD) of three independent experiments. Source data are provided as a Source Data file.

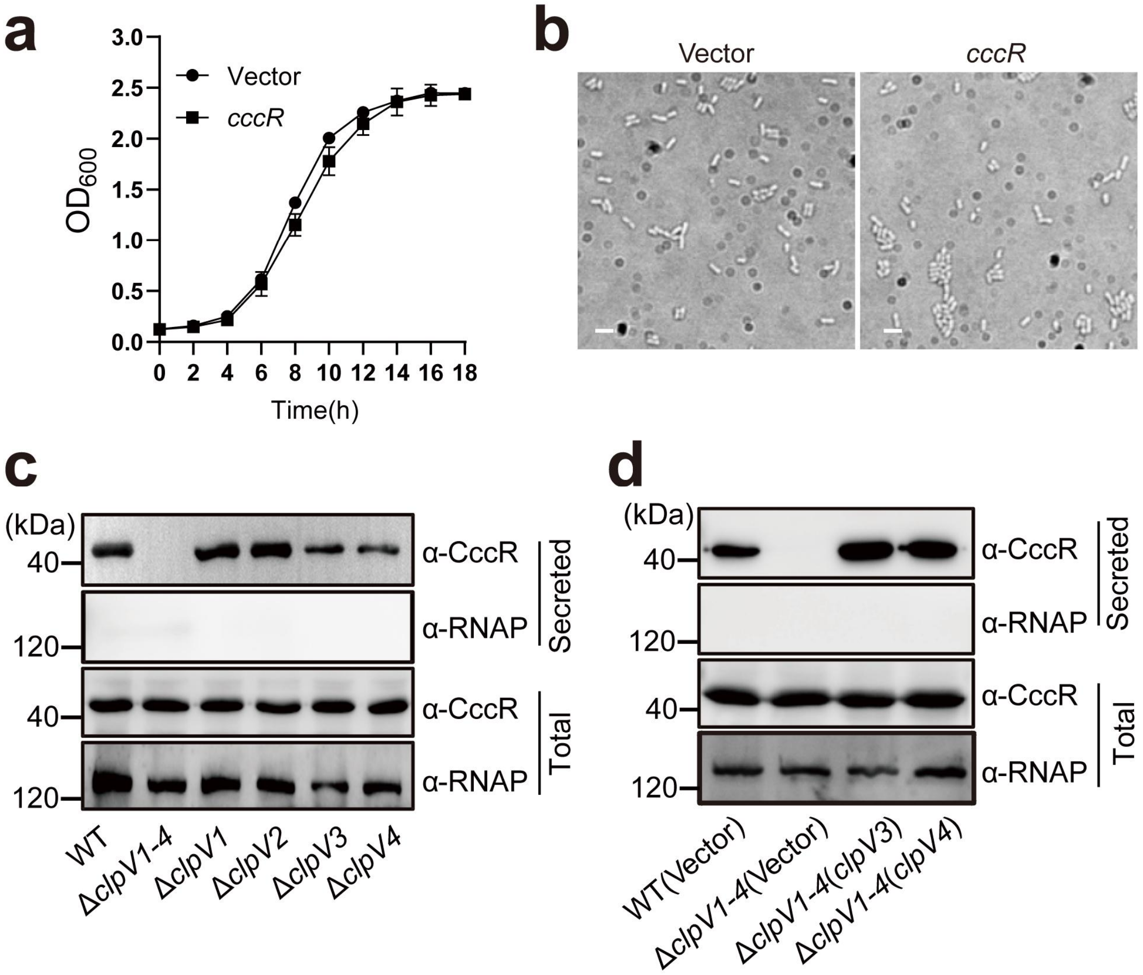

Supplementary Fig. 2 | CccR is secreted by T6SS-3 and T6SS-4.

a-b, Effects of *cccR* expression on *Yptb* growth. Growth curve (**a**) and microscopic analysis (**b**) of *Yptb* cells overexpressing *cccR* with the pME6032 vector. Data in **a** are presented as the mean ± standard deviation (SD) of three independent experiments. **c-d**, CccR is secreted by T6SS-3 and T6SS-4. A pME6032 vector expressing *cccR* was introduced into indicated *Yptb* strains. Total cell pellet (Total proteins) and secreted proteins in the culture supernatant (Secreted proteins) were isolated and probed for the presence of the CccR protein. Cytosolic RNA polymerase (RNAP) was probed as a control. The blots shown are representative of three separate experiments with similar results. The blots shown are representative of three separate experiments with similar results. Source data are provided as a Source Data file.

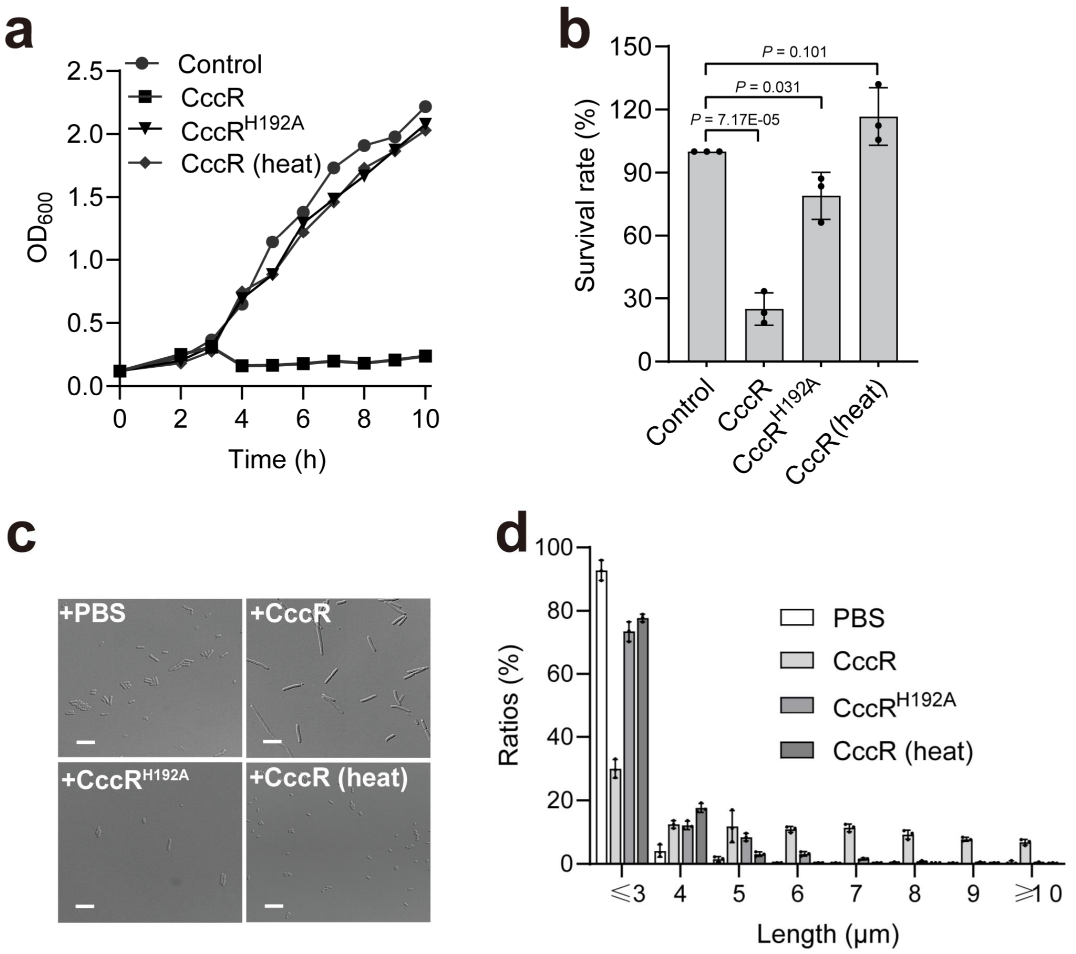

Supplementary Fig. 3 | Exogenously provided CccR protein affects the growth and morphology of *E. coli* cells.

a, Exogenously provided CccR inhibits the growth of *E. coli* cells. Growth curves of *E. coli* BL21(DE3) in the presence of exogenously provided CccR proteins were obtained by measuring OD_{600} at 1 h intervals, with PBS as a control. Data are presented as the mean ± standard deviation (SD) of three independent experiments. **b**, Toxicity assays of exogenously provided CccR proteins to *E. coli*. BL21(DE3) cells were treated with CccR, heat inactivated CccR (heat) or CccR^{H192A} proteins (250 ng/mL) for 6 h, and the viability of cells was determined by counting the CFU after treatment. **c**, Exogenously provided CccR induces filamentation of *E. coli* cells. Representative micrographs of BL21(DE3) cells after coincubation with CccR, CccR (heat) or CccR^{H192A} proteins (250 ng/mL) for 6 h. The images shown are representative of three separate experiments with similar results. Scale bar, 10 μm. **d**, Distribution of CccR treated *E. coli* cells with different lengths. The length of 500 cells was measured from each of three samples, and their distribution was plotted. Data shown are representative from three independent experiments. Data in **b** and **d** are presented as the mean ± standard deviation (SD) of three independent experiments. P values from all data were determined using a two-sided, unpaired Student's t-test, and differences were considered significant at $P < 0.05$. Source data are provided as a Source Data file.

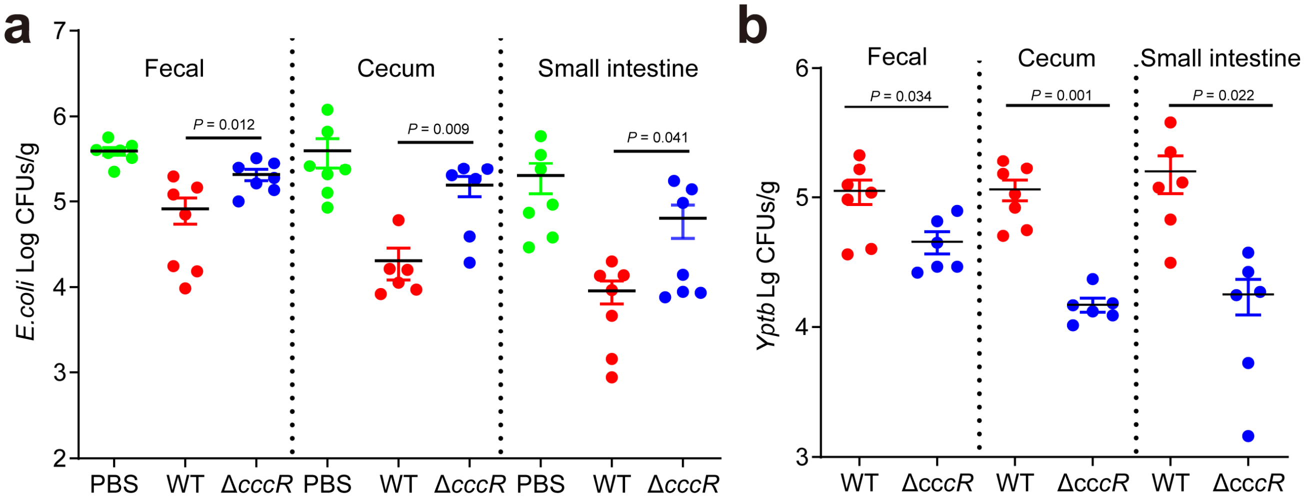

Supplementary Fig. 4 | CccR mediates interspecies competition *in vivo*.

Streptomycin-treated mice (n= 6-9) were colonized with 5×10^8 CFUs of *E. coli* DH5α cells for 24 h and then challenged with 5×10^8 CFUs of *Yptb* WT, Δ*cccR* or PBS buffer. Animals were sacrificed 24 h after the challenge, and survival of *E. coli* (**a**) and *Yptb* (**b**) in the cecum and small intestine were counted. Data are represented as mean values ± standard deviation (SD) of three biological replicates, each of which was performed in three technical replicates. *P* values from all data were determined using a two-sided, unpaired Student's *t*-test, and differences were considered significant at $P < 0.05$. Source data are provided as a Source Data file.

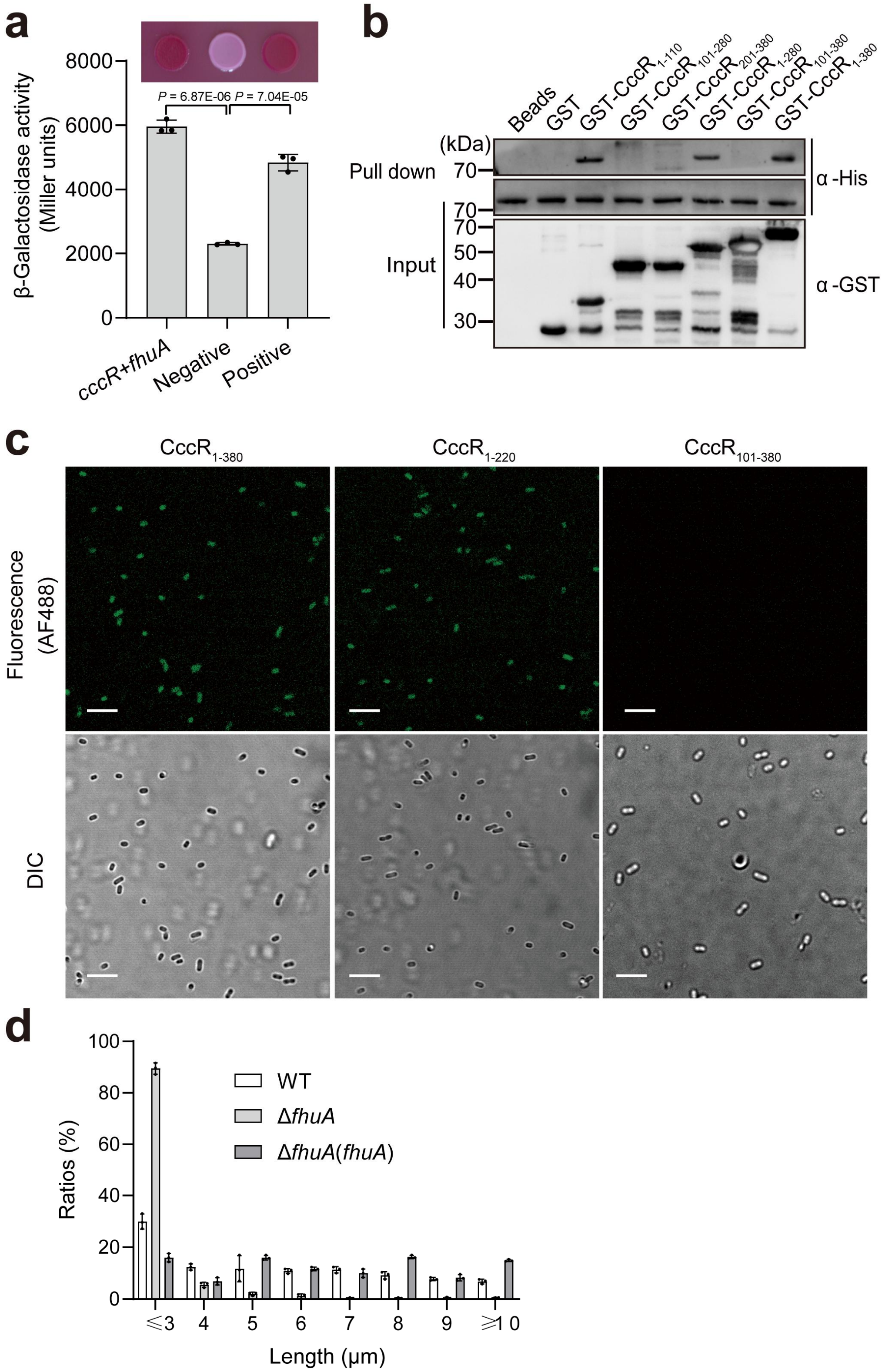

Supplementary Fig. 5 | The N-terminus 100 aa of CccR is required for target cell entry.

a, Interaction between CccR and FhuA was verified with bacterial two-hybrid assay. Interactions were assessed using MacConkey maltose plates (upper) and the β-galactosidase assay (lower). Data are presented as the mean ± standard deviation (SD) of three independent experiments. *P* values from all data were determined using a two-sided, unpaired Student's *t*-test, and differences were

considered significant at $P < 0.05$. **b**, Direct binding between His_6-FhuA and indicated GST-CccR truncations was detected with *in vitro* GST pull-down assay. The protein complexes captured on glutathione beads were detected using western blotting. The blots shown are representative of three separate experiments with similar results. **c**, Fluorescence labeling of *E. coli* BL21(DE3) cells with indicated CccR truncations conjugated with AF488. Note that $CccR_{101-380}$ shows no entry, while $CccR_{1-380}$ and $CccR_{1-220}$ enter into *E. coli* cells. The images shown in are representative of three separate experiments with similar results. Scale bar, 10 µm. **d,** Distribution of CccR treated *E. coli* BL21(DE3) cells with different lengths. The length of 500 cells was measured from each of three samples, and their distribution was plotted. Data are presented as the mean ± standard deviation (SD) of three independent experiments. Source data are provided as a Source Data file.

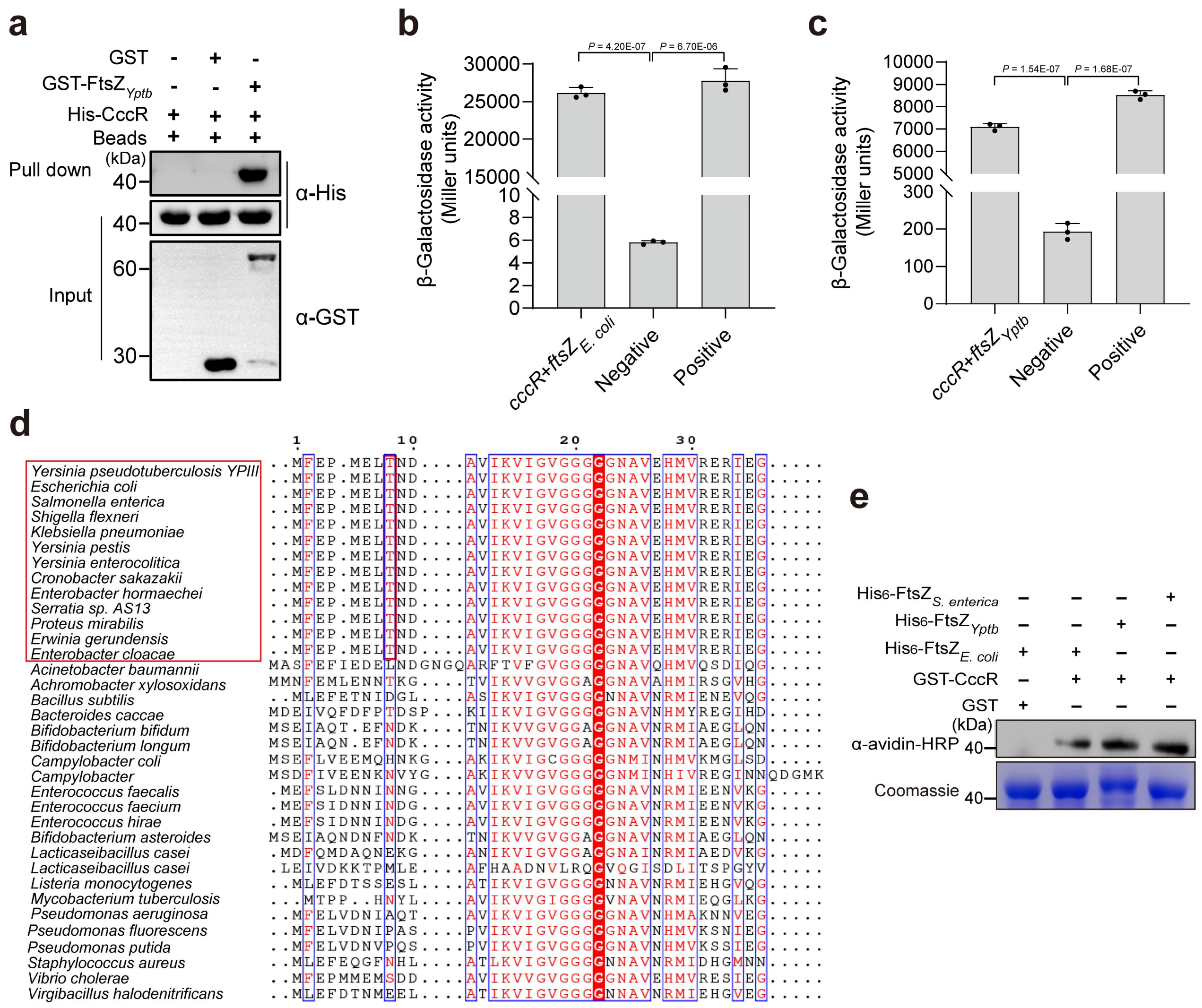

Supplementary Fig. 6 | CccR AMPylates FtsZ from different strains at Thr[8].

a, Direct interaction between His_6-CccR and GST-$FtsZ_{Yptb}$ verified with an *in vitro* GST pull-down assay. The blots shown are representative of three separate experiments with similar results. **b-c**, Interaction between CccR and FtsZ confirmed using bacterial two-hybrid assay. Interactions between CccR and FtsZ of *E. coli* (**b**) and *Yptb* (**c**) were assessed with the β-galactosidase assay. Data in **b** and **c** are presented as the mean ± standard deviation (SD) of three independent experiments. *P* values from all data were determined using a two-sided, unpaired Student's *t*-test, and differences were considered significant at $P < 0.05$. **d,** Sequence alignment of FtsZ proteins from different intestinal bacterial strains. Thr[8] is relatively conserve in bacteria of Enterobacteriaceae but not in other families. **e**, Representative blot using avidin-HRP to detect biotinylated proteins following incubation of FtsZ proteins from indicated strains with bio-17-ATP and CccR proteins. Coomassie bright blue staining is shown as a loading control. The blots shown are representative of three separate experiments with similar results. Source data are provided as a Source Data file.

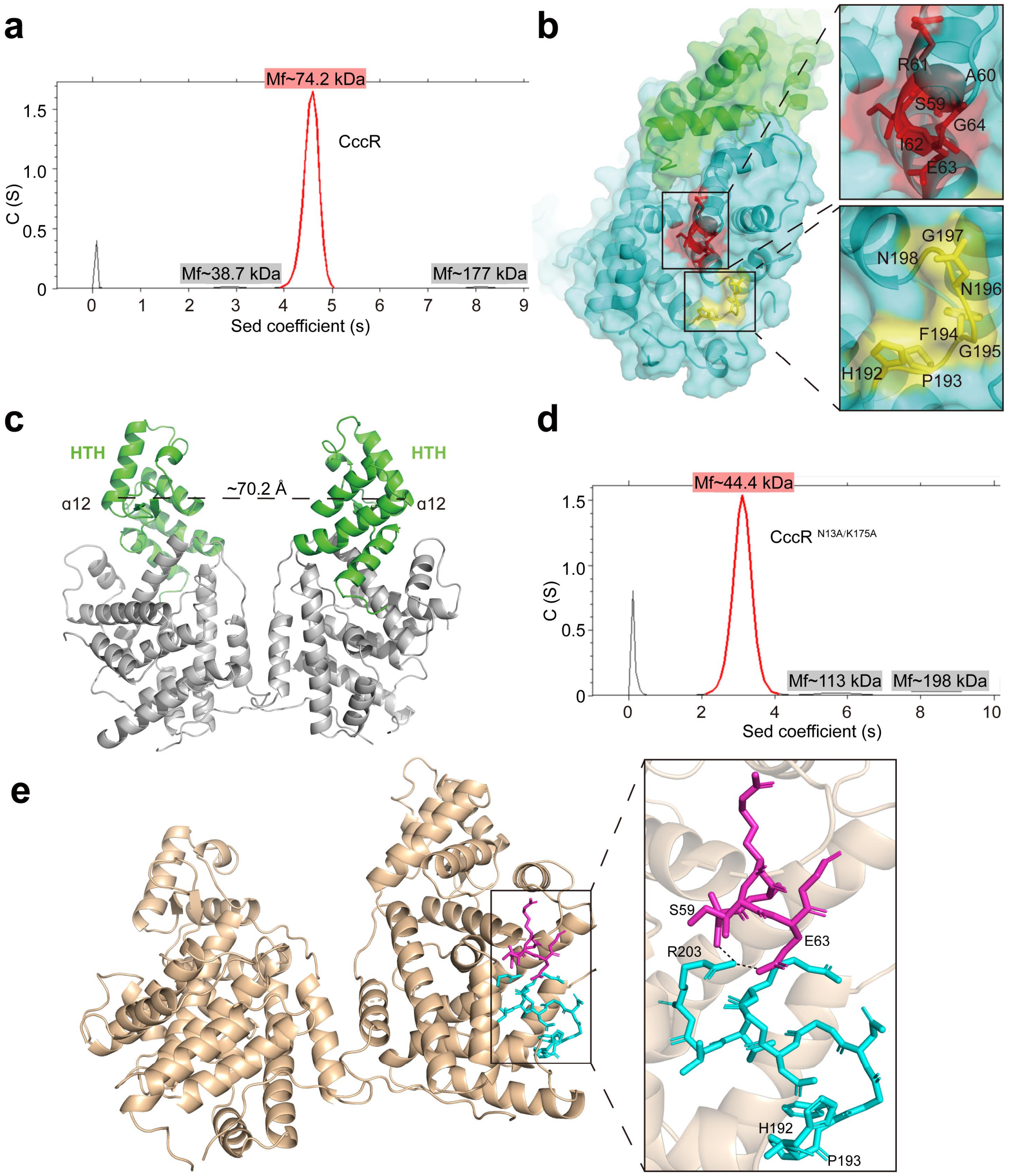

Supplementary Fig. 7 | Overall structure of CccR.

a, Analytical ultracentrifugation profile (AUC) showing a single monodisperse peak at ~74.2 kDa, corresponding to two times the theoretical molecular mass of the CccR monomer. **b**, Location of FIC motif (H_{192}PFGNGNGRTVR$_{203}$, yellow) and the predicted inhibitory helix (red) in CccR. **c**, The distance between HTH domain in the dimer is indicated. The distance between the α12 helix of the two HTH domains in the dimer are approximately 70.2 Å. **d**, AUC of CccR$^{N13A/K175A}$, showing a single monodisperse peak at ~44.4 kDa, corresponding to the theoretical molecular mass of the CccR monomer. **e**, The relationship between the inhibitory helix and the active center. The S_{59} and E_{63} residue of inhibitory helix (purple) form two potential hydrogen bonds with R_{203} (blue) in the active center to prevent binding of the ATP substrate for modification.

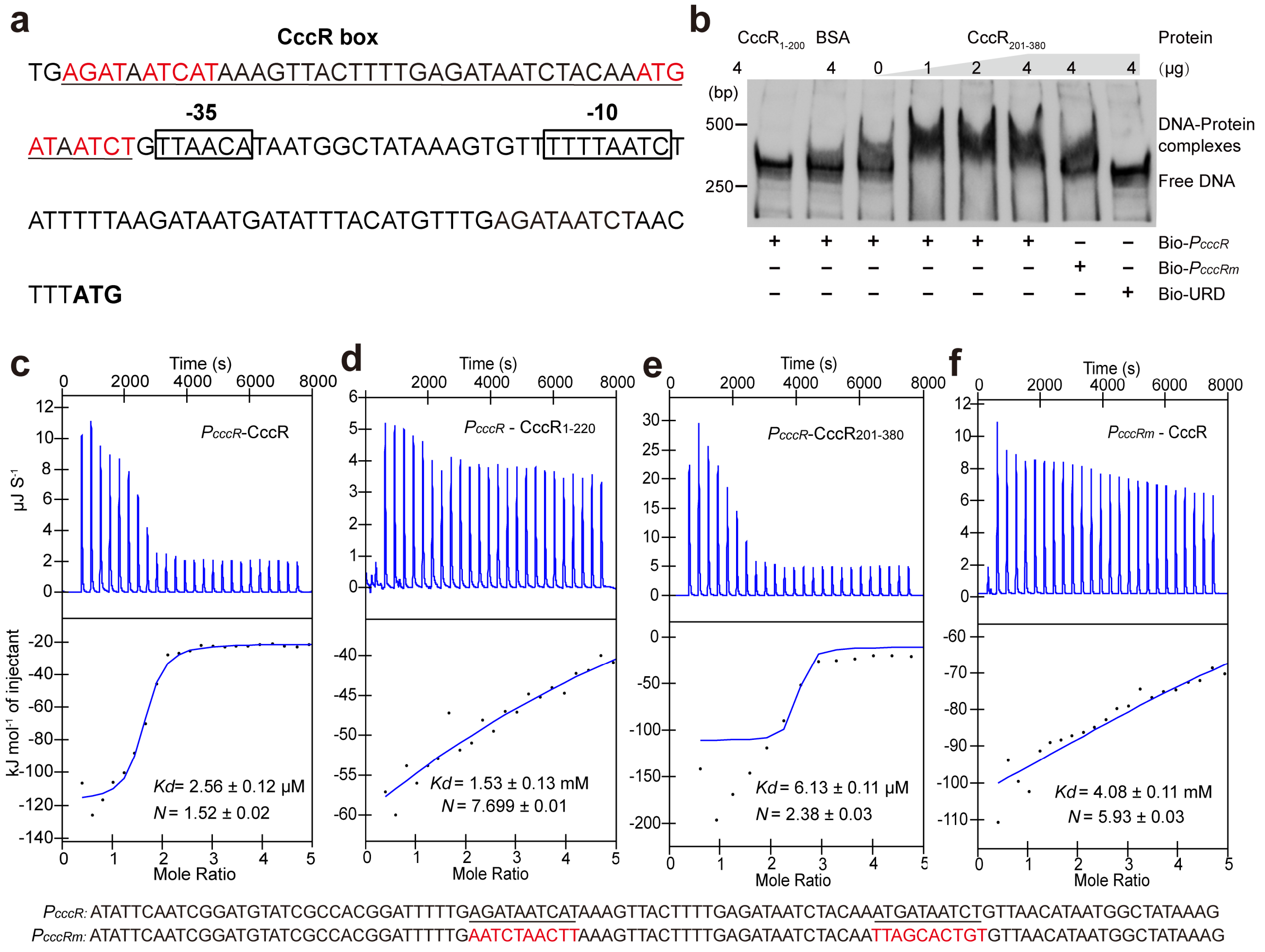

Supplementary Fig. 8 | CccR binds to its own promoter.

a, Nucleotide sequence of the *cccR* promoter region. The CccR-binding site identified by DNase I footprinting analysis is underlined, with the palindrome sequences shown in red. Putative −35 and −10 elements of the *cccR* promoter are boxed. The ATG start codon of the *cccR* ORF is shown in bold. **b**, Binding of CccR to the *cccR* promoter examined by EMSA. EMSA was performed to analyze the interactions between His_6-CccR and the *cccR* promoter (P_{cccR}), the *cccR* promoter with mutations in CccR binding site (P_{cccRm}), or unrelated DNA fragment (URD). $CccR_{1\text{-}200}$ (4 μg), increasing amounts of $CccR_{201\text{-}380}$ (0, 1, 2 and 4 μg) and 20 nM DNA fragments were used in each reaction. The blots shown are representative of three separate experiments with similar results. **c-f**, The ITC fitting results of CccR proteins with *cccR* promoter probe. The thermodynamic data were collected from injections of P_{cccR} into CccR (**c**), $CccR_{1\text{-}220}$ (**d**), $CccR_{201\text{-}380}$ (**e**), or P_{cccRm} into CccR (**f**), respectively, and the binding isotherm was fitted to a one-site binding model after subtraction of blank titration heats. *Kd* and complex stoichiometry (*n*) are presented as mean ± standard deviation (SD) of three independent experiments. Source data are provided as a Source Data file.

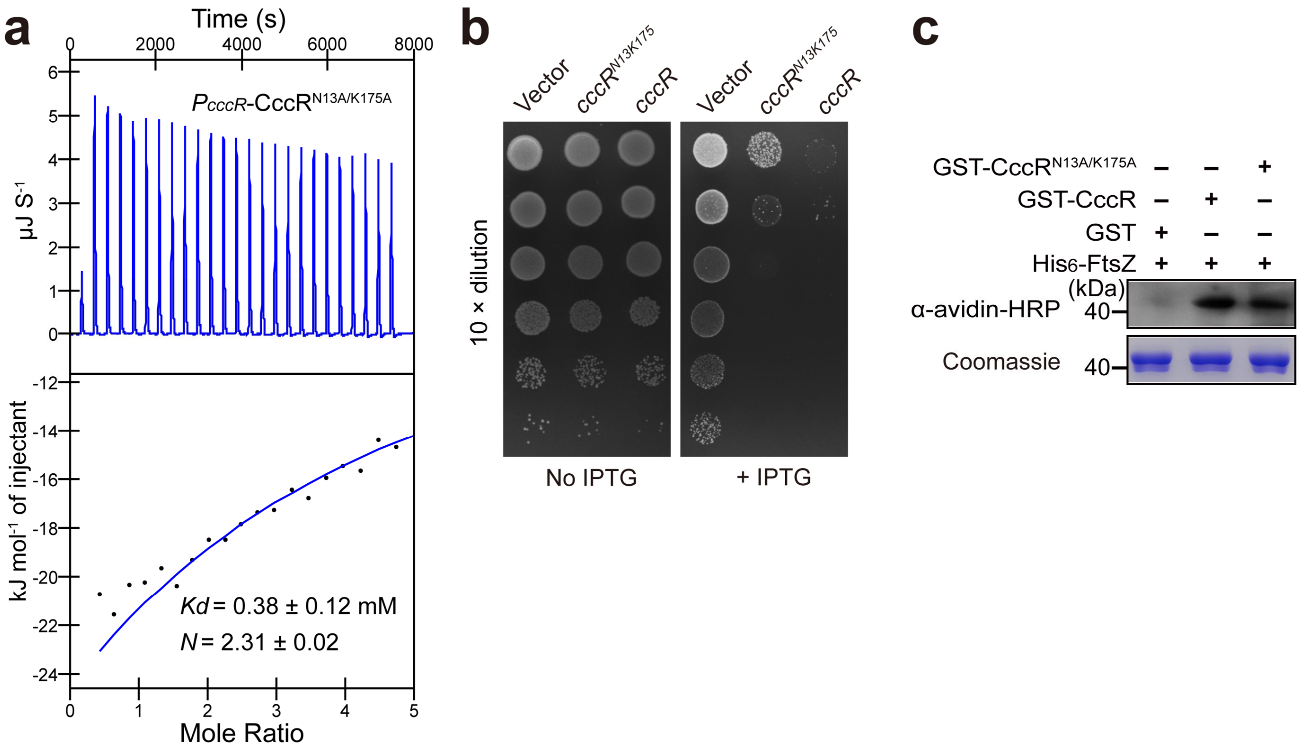

Supplementary Fig. 9 | Effect of dimerization plane mutation on CccR function.

a, The ITC fitting results of CccR$^{N13A/K175A}$ protein with *cccR* promoter probe. The thermodynamic data were collected from injections of P_{cccR} probe into CccR$^{N13A/K175A}$, and the binding isotherm was fitted to a one-site binding model after subtraction of blank titration heats. *Kd* and complex stoichiometry (*n*) are presented as mean ± standard deviation (SD) of three independent experiments. **b**, CccR$^{N13A/K175A}$ is toxic to *E. coli*. Growth of *E. coli* BL21(DE3) cells containing a vector control or a vector expressing CccR or CccR$^{N13A/K175A}$ under noninducing (no IPTG) or inducing (100 μM IPTG) conditions. The results shown are representative of three separate experiments with similar results. **c**, Representative blot using avidin-HRP to detect biotinylated proteins following incubation of *E. coli* FtsZ with bio-17-ATP and CccR or CccR$^{N13A/K175A}$ proteins. Coomassie bright blue staining is shown as a loading control. The blots shown are representative of three separate experiments with similar results. Source data are provided as a Source Data file.

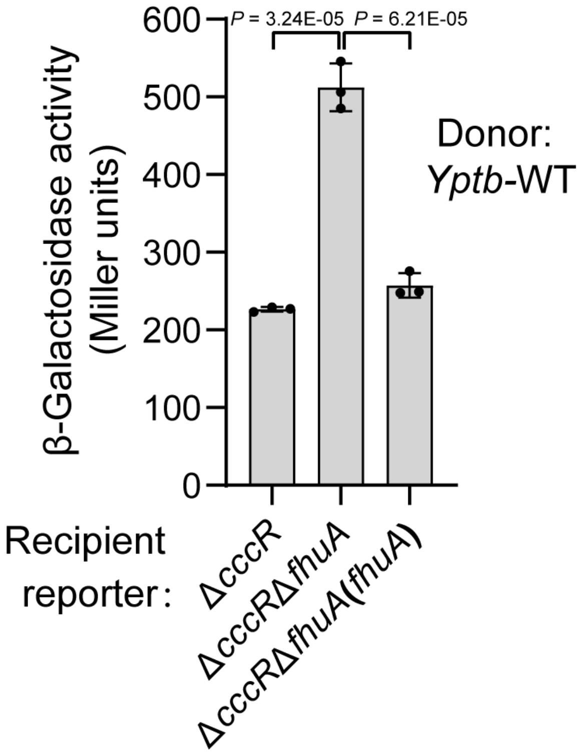

Supplementary Fig. 10 | FhuA is required for delivered CccR to exert transcriptional regulator activity in kin cells.

Effects of CccR delivered from the *Yptb* WT donor on P_{cccR} promoter activity in the Δ*cccR*, Δ*cccR*Δ*fhuA* and Δ*cccR*Δ*fhuA*(*fhuA*) recipient reporter strains were determined with β-galactosidase assays. Transwells were used to separate the *Yptb* WT donor and indicated recipient reporter strains, and the LacZ activity in the recipient reporter strains was detected after coincubation at 26°C for 12 h. Data are presented as the mean ± standard deviation (SD) of three independent experiments. *P* values were determined using a two-sided, unpaired Student's *t*-test, and differences were considered significant at $P < 0.05$. Source data are provided as a Source Data file.

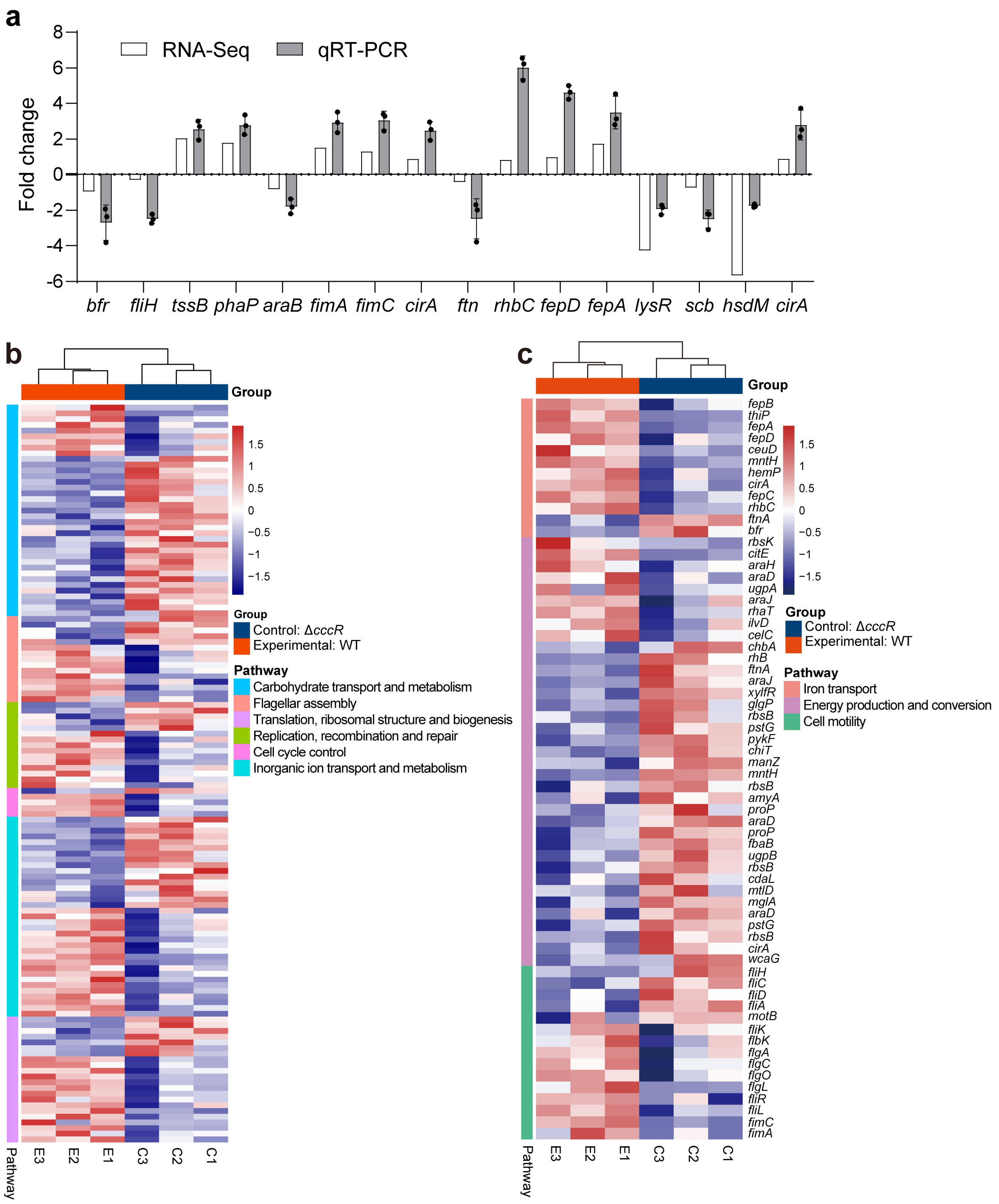

Supplementary Fig. 11 | Delivered CccR regulates gene expression in kin cells.

a, Validation of RNA-seq results via qRT-PCR. Sixteen representative genes were chosen to validate the RNA-seq data by qRT-PCR. CccR^{H192A} (200 ng/mL) was added to the Δ*cccR* culture when OD_{600}=0.5 and continue incubation at 26°C until OD_{600}=1.0. RNA was extracted to evaluate the transcription of genes by qRT-PCR. Equal volume of PBS buffer was served as the control. The fold

change means multiple of $CccR^{H192A}$ treated over $CccR^{H192A}$ untreated samples. White bars represent RNA-seq data. Data are presented as the mean ± standard deviation (SD) of three independent experiments. **(b-c)** Comparative transcriptomic analysis of Δ*cccR* cells coincubated with *Yptb* WT or Δ*cccR*, respectively, in separated wells of the Transwell system. Heat map of RPKMs (reads per kilobase of transcript per million reads mapped) for the genes that were expressed in Δ*cccR* cells coincubated with *Yptb* WT (E1-E3) compared to genes expressed in Δ*cccR* cells coincubated with Δ*cccR* (C1-C3). Shading indicates low (blue) and high (red) RPKM values for each triplicate samples of each strain. Genes are arranged in pathways. Three replicates were done for each experiment. Source data are provided as a Source Data file.

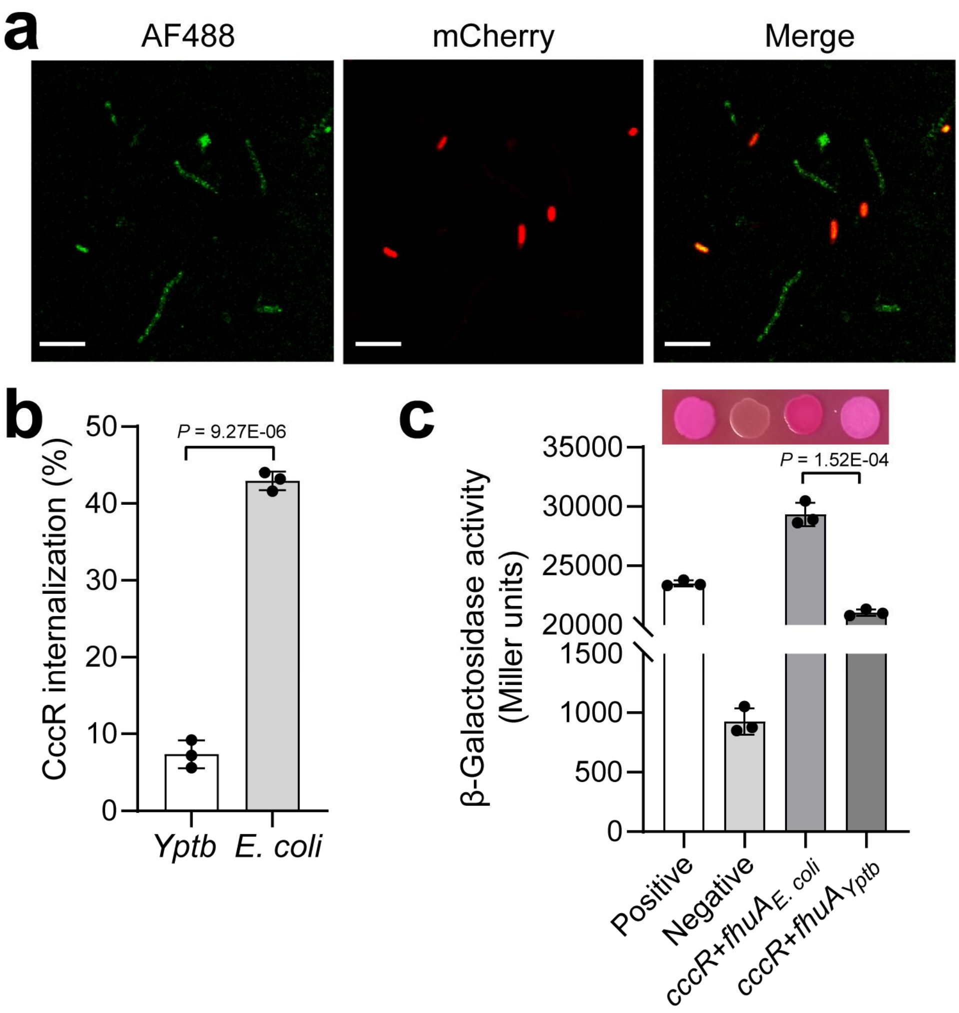

Supplementary Fig. 12 | CccR exhibited higher affinity to *E. coli* FhuA than to *Yptb* FhuA and enter *E. coli* cells more efficient than *Yptb* cells.

a-b, Competitive internalization of CccR protein by *E. coli* and *Yptb* cells. Unlabeled *E. coli* and mCherry-labeled *Yptb* cells were mixed in a 1:1 ratio and incubated with AF488-conjugated CccR (Green) for 1 h at 30 °C. After 3 times wash with PBS, the internalization of CccR in different cells were observed by confocal microscopy (**a**), and the percentages of *E. coli* and *Yptb* cells that exhibited green fluorescence (indicating CccR internalization) were quantified (**b**). The images shown are representative of three separate experiments with similar results. Scale bar, 10 μm. **c**, Comparison of the interaction of CccR with FhuA from *E. coli* and *Yptb* with bacterial two-hybrid assay. Interactions were assessed using MacConkey maltose plates (upper) and the β-galactosidase assay (lower). Data in **b** and **c** are presented as the mean ± standard deviation (SD) of three independent experiments. *P* values from all data were determined using a two-sided, unpaired Student's *t*-test, and differences were considered significant at $P < 0.05$. Source data are provided as a Source Data file.

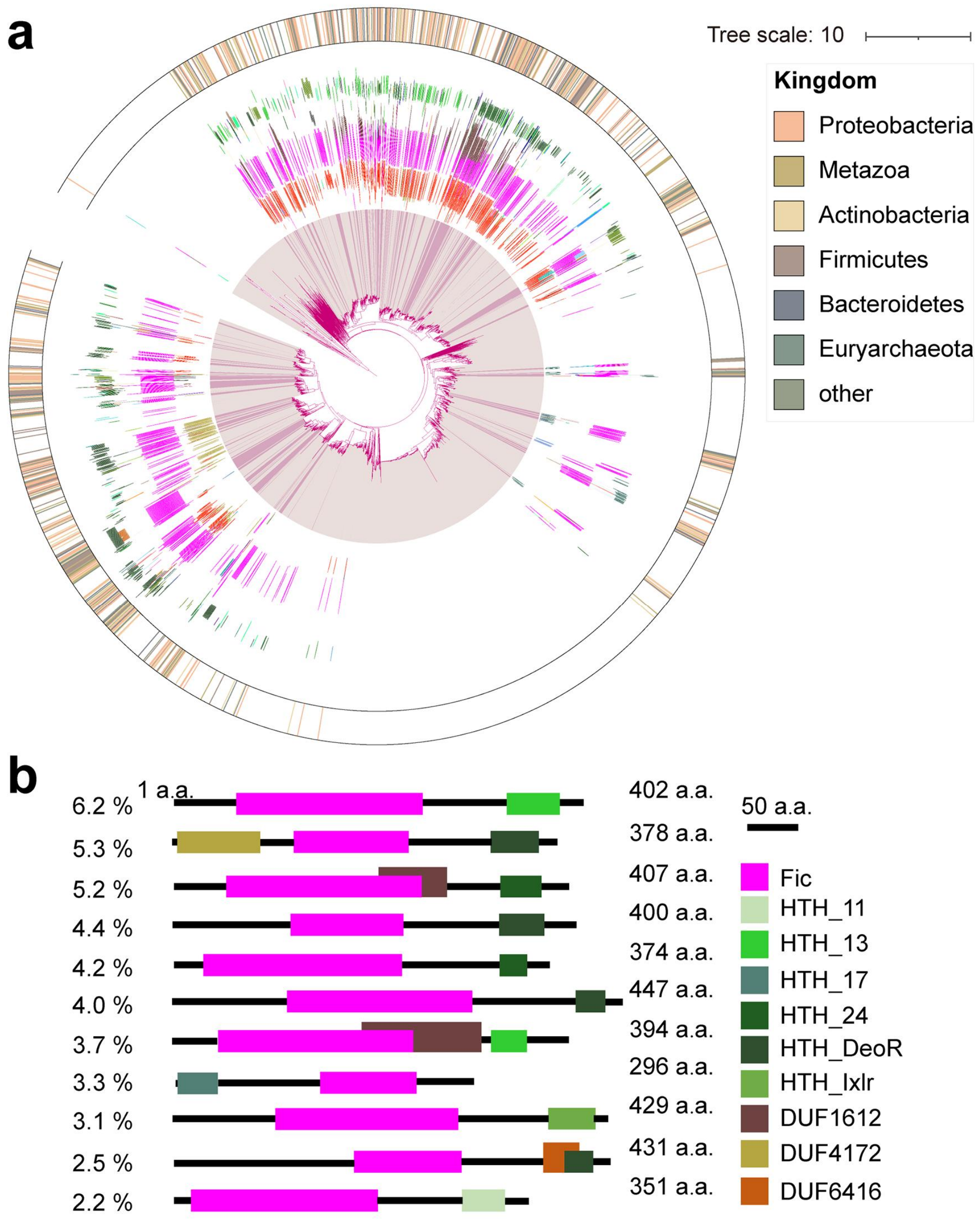

Supplementary Fig. 13 | The wide distribution of HTH-containing FIC proteins in prokaryotes and eukaryotes.

a, Evolutionary tree of CccR and 3040 homologous proteins. A total of 3040 CccR homologs were identified by orthologs search from the KEGG database among which 731 proteins contain both the FIC domain and HTH domain, and are widely distributed in prokaryotes and eukaryotes. All identified FIC protein sequences were combined and compared using muscle v3.8.1551 and a genealogical tree was constructed based on the FastTree version 2.1.10. The results of the phylogenetic tree were drawn and modified using iTOL online analytical. **b**, The domain architecture of HTH-containing FIC homologs. 259 types of domain combinations were observed for HTH-containing FIC homologs and the top 11 domain combinations (accounting for 44% of the 731 HTH-containing FIC homologs) were shown. Magenta represents FIC domains and other colors represent the other domains.

Supplementary Table 1 | Data collection and refinement statistics.

**Data collection**	**SeMet FIC**
Wavelength (Å)	0.9792
Space group	C222
Cell dimensions	
a, b, c (Å)	115.46, 282.77, 73.57
α, β, γ (°)	90.00, 90.00, 90.00
Resolution (Å)	141.38-2.78 (2.85-2.78)
No. of reflections	30803(3083)
Rmerge	0.186 (4.592)
I/σI	10.7(0.7)
Completeness (%)	100 (99.67)
Redundancy	13.2 (13.4)
Refinement	
Resolution (Å)	33.86-2.78 (2.88-2.78)
No. reflections	30713 (3029)
Rwork/Rfree (%)	22.91(38.85)/25.74 (37.57)
Total no. of atoms	5732
Ramachandran plot	
Favoured (%)	96.06
Allowed (%)	3.80
Outliers (%)	0.14
