# Supplementary material for: A secreted effector with a dual role as a toxin and as a transcriptional factor
Source: Nat Commun. 2022 Dec 16;13:7779. doi: 10.1038/s41467-022-35522-9 (PMC9755527; doi:10.1038/s41467-022-35522-9)
Supplement: Supplementary file 3 — Description of Additional Supplementary Files [file 41467_2022_35522_MOESM3_ESM.pdf]

Description of Additional Supplementary Files

File Name: Supplementary Data 1

Description: Specific pathways regulated by delivered CccR in Yptb.

File Name: Supplementary Data 2

Description: Bacterial strains and plasmids used in this study.

File Name: Supplementary Data 3

Description: Primers used in this study.
