# Supplementary material for: A secreted effector with a dual role as a toxin and as a transcriptional factor
Source: Nat Commun. 2022 Dec 16;13:7779. doi: 10.1038/s41467-022-35522-9 (PMC9755527; doi:10.1038/s41467-022-35522-9)
Supplement: Supplementary file 7 — Reporting Summary [file 41467_2022_35522_MOESM7_ESM.pdf]

nature portfolio

Corresponding author(s): Xihui Shen and Songying Ouyang

Last updated by author(s): Dec 4, 2022

Reporting Summary

Nature Portfolio wishes to improve the reproducibility of the work that we publish. This form provides structure for consistency and transparency in reporting. For further information on Nature Portfolio policies, see our Editorial Policies and the Editorial Policy Checklist.

Statistics

For all statistical analyses, confirm that the following items are present in the figure legend, table legend, main text, or Methods section.

n/a	Confirmed	
☐	☒	The exact sample size (*n*) for each experimental group/condition, given as a discrete number and unit of measurement
☐	☒	A statement on whether measurements were taken from distinct samples or whether the same sample was measured repeatedly
☐	☒	The statistical test(s) used AND whether they are one- or two-sided<br>*Only common tests should be described solely by name; describe more complex techniques in the Methods section.*
☒	☐	A description of all covariates tested
☒	☐	A description of any assumptions or corrections, such as tests of normality and adjustment for multiple comparisons
☐	☒	A full description of the statistical parameters including central tendency (e.g. means) or other basic estimates (e.g. regression coefficient) AND variation (e.g. standard deviation) or associated estimates of uncertainty (e.g. confidence intervals)
☐	☒	For null hypothesis testing, the test statistic (e.g. *F*, *t*, *r*) with confidence intervals, effect sizes, degrees of freedom and *P* value noted<br>*Give P values as exact values whenever suitable.*
☒	☐	For Bayesian analysis, information on the choice of priors and Markov chain Monte Carlo settings
☒	☐	For hierarchical and complex designs, identification of the appropriate level for tests and full reporting of outcomes
☒	☐	Estimates of effect sizes (e.g. Cohen's *d*, Pearson's *r*), indicating how they were calculated

Our web collection on statistics for biologists contains articles on many of the points above.

Software and code

Policy information about availability of computer code

Data collection	Bright-field cell omages were captured using A; Fluoresence observation of cells were measureed by B; Isothermal titration calorimetry assays were collected by TA Advantage 11.0.0; Chemical fluorescence image data were collected by Tanon 5200Multi Chemiluminescence imager; The absorbance were measured by Thermo Fisher Scientific NanoDrop 2000; Crystal data were collected by HKL2000; Sedimentation coefficient was obtained using Sedfit; Gene or genome, protein, structure information is obtained from the KEGG, Uniport, and PDB database, respectively.
Data analysis	Statistical analysis has been performed using GraphPad Prism 9.0 software; The ITC data were analyzed using the NanoAnalyze_3.4; Gel images were analyzed using Gel Analysis 4.2; Gel quantitative analysis was performed by ImageJ v1.48; Homologous proteins were compared using software muscle v3.8.1551; evolutionary tree was constructed using FastTree version 2.1.10; Image analysis were performed using Imaris x64 9.0.1; Protein structure analysis were performed using PyMOL software; Mass spectrometry were analyzed using Mascot search engine v.2.5.1.

For manuscripts utilizing custom algorithms or software that are central to the research but not yet described in published literature, software must be made available to editors and reviewers. We strongly encourage code deposition in a community repository (e.g. GitHub). See the Nature Portfolio guidelines for submitting code & software for further information.

Data

Policy information about availability of data

All manuscripts must include a data availability statement. This statement should provide the following information, where applicable:

- Accession codes, unique identifiers, or web links for publicly available datasets
- A description of any restrictions on data availability
- For clinical datasets or third party data, please ensure that the statement adheres to our policy

The atomic coordinates and structure factors of the CccR have been deposited in the Protein Data Bank (PDB) under accession code 7XUX (https://www.rcsb.org/structure/unreleased/7XUX). RNA-seq raw FASTQ files for the RNA-seq libraries have been deposited in the NCBI Sequence Read Archive (SRA) under the code BioProject accession PRJNA905726 (https://www.ncbi.nlm.nih.gov/bioproject/PRJNA905726). All the other data that support the findings of this study are available within the paper and its Supplementary Information and Supplementary Data, or from the corresponding authors upon request. 7.The source data underlying Figs. 1e-f, 2a, b, d, f, 3a, c, d, e, and 5a, c, d, e and Supplementary Figs. 1a, c, 2a, c, d, 3a, b, d, 4a-b, 5a, b, d, 6a-c, d, 8b-f, 9c, 10, 11a and 12b-c are provided as a Source Data file. All the other data that support the findings of this study are available within the paper and its Supplementary Information and Supplementary Data or from the corresponding authors upon request. Source data are provided with this paper.

Field-specific reporting

Please select the one below that is the best fit for your research. If you are not sure, read the appropriate sections before making your selection.

☒ Life sciences ☐ Behavioural & social sciences ☐ Ecological, evolutionary & environmental sciences

For a reference copy of the document with all sections, see nature.com/documents/nr-reporting-summary-flat.pdf

Life sciences study design

All studies must disclose on these points even when the disclosure is negative.

Sample size	For in vitro experiments, sample sizes were determined without statistical measures, but based on widely used sizes in relevant publications (Zhang et al. Nat. Commun. 2022, 13:6684; Zhang et al. Nat. Commun. 2020, 11:5371; Gan et al. Nat Microbiol. 2019, 4:134-143; Lin et al. Nat. Commun. 2017, 8:14888) to ensure that it will be appropriate for statistical analysis. Mouse in vivo competition assays were conducted twice with at least 6 mice ($n \geq 6$) in each infection group, and the combined data for the two experiments were used for statistical analysis, according to the method by Jiang et al. (Nat. Commun. 2021, 12:879).
Data exclusions	No data were excluded from the analyses.
Replication	Mouse in vivo competition assays were performed in two independent experiments, with 6–7 mice/group/experiment. All other experiments were performed for at least 3 times. All the attempts at replication were successful.
Randomization	All animals used were age, sex and vendor matched. Animals were randomly allocated to each group. For experiments other than those involving animals, randomization is not applicable as it employed bacterial strains.
Blinding	Investigators were not blinded during data collection or analysis since there was not group allocation.

Reporting for specific materials, systems and methods

We require information from authors about some types of materials, experimental systems and methods used in many studies. Here, indicate whether each material, system or method listed is relevant to your study. If you are not sure if a list item applies to your research, read the appropriate section before selecting a response.

Materials & experimental systems

n/a	Involved in the study	
☐	☒	Antibodies
☒	☐	Eukaryotic cell lines
☒	☐	Palaeontology and archaeology
☐	☒	Animals and other organisms
☒	☐	Human research participants
☒	☐	Clinical data
☒	☐	Dual use research of concern

Methods

n/a	Involved in the study	
☒	☐	ChIP-seq
☒	☐	Flow cytometry
☒	☐	MRI-based neuroimaging

Antibodies

Antibodies used	Antibodies used in this study include anti-CccR (Laboratory preparation), 1:1000; rabbit anti-GST (Santa Cruz, cat# 53909), 1:1000;

	rabbit anti-RNAP (Santa Cruz, cat# sc-56766), 1:5000; mouse anti-His (Santa Cruz, cat# sc-8036), 1:5000; 1:5,000; Goat anti-Mouse horseradish peroxidase-conjugated secondary antibodies (DIYIBIO, China, cat# DY60203), 1:10,000; Goat anti-Rabbit horseradish peroxidase-conjugated secondary antibodies (DIYIBIO, China, cat# DY60202), 1:10,000.
Validation	All antibodies used in this study are commercially avaiable and have been validated for the apecificity by the manufacturers as follows.<br>RNA pol β (8RB13) is a mouse monoclonal antibody raised against RNA pol β of E. coli origin. RNA pol β (8RB13) is recommended for detection of RNA pol β of E. coli origin by Western Blot (starting dilution 1:200), https://datasheets.scbt.com/sc-56766.pdf.<br>His-probe (H-3) is a mouse monoclonal antibody raised against a His tagged recombinant protein, His-probe (H-3) is recommended for detection of fusion proteins encoded by polyhistidine expression vectors origin by Western Blot (starting dilution 1:200), https://datasheets.scbt.com/sc-8036.pdf.<br>GST (1E5) is a mouse monoclonal antibody raised against recombinant Glutathione S-transferase.GST (1E5) is recommended for detection of GST by Western Blotting (starting dilution 1:200, dilution range 1:100-1:1000), https://datasheets.scbt.com/sc-53909.pdf.<br>The antibodies were validated by the supplier (see website) and also in our laboratory using Western Blot assays in Yersinia pseudotuberculosis and are cited in our labs previous publications.<br>-Xu L et al. Inhibition of host vacuolar H+-ATPase activity by a Legionella pneumophila effector. PLoS Pathog. (2010).<br>-Wang T et al. Type VI Secretion System Transports Zn2+ to Combat Multiple Stresses and Host Immunity. PLoS Pathog. (2015).<br>-Si M et al. Manganese scavenging and oxidative stress response mediated by type VI secretion system in Burkholderia thailandensis. Proc Natl Acad Sci U S A. (2017).

Animals and other organisms

Policy information about studies involving animals; ARRIVE guidelines recommended for reporting animal research

Laboratory animals	Six-week-old female mice (BALB/c) were purchased from the central animal laboratory of Xi'An JiaoTong University (Xi'an, China) and kept in a temperature (24±2°C), 50±10% humidity, air flow of 35 exchanges and light-controlled room (12 h light, 12 h darkness) with free access to food and water.
Wild animals	No wild animals were used in the study.
Field-collected samples	No field-collected samples were used in the study.
Ethics oversight	All mouse experimental procedures were performed in accordance with the Regulations for the Administration of Affairs Concerning Experimental Animals approved by the State Council of People's Republic of China. The protocol was approved by the Animal Welfare and Research Ethics Committee of Northwest A&F University (protocol number: NWAFUSM2018001).

Note that full information on the approval of the study protocol must also be provided in the manuscript.
